# Dynamic changes and prognostic value of glutathione S-transferase alpha in mild cognitive impairment and Alzheimer’s disease

**DOI:** 10.3389/fnagi.2024.1517613

**Published:** 2024-12-23

**Authors:** Yangyang Tang, Ni Li, Linyan Dai, Xingsheng Wang, Xia Lai

**Affiliations:** Department of Geriatrics, Chongqing University Central Hospital, Chongqing Emergency Medical Center, Chongqing, China

**Keywords:** Alzheimer’s disease, mild cognitive impairment, GSTα, blood markers, MCI (mild cognitive impairment)

## Abstract

**Objectives:**

Glutathione S-transferase alpha (GSTα) is an important antioxidant enzyme closely associated with the onset and progression of neurodegenerative diseases. The alterations in GSTα protein levels associated with Alzheimer’s disease and their impact on cognitive abilities remain unclear. Thus, investigating the fluctuations of GSTα protein levels in mild cognitive impairment (MCI) and Alzheimer’s disease (AD) is essential.

**Methods:**

DATA were enrolled from the Alzheimer’s Disease Neuroimaging Initiative (ADNI) database, and we studied healthy individuals (as controls, a total of 54), patients with mild cognitive impairment (345), and patients with Alzheimer’s disease (96) A one-year follow-up was conducted to collect data on the dynamic changes of GSTα protein levels in plasma and primary information data, and to analyze the correlation between the changes in GSTα protein levels before and after the follow-up and cognitive function and its predictive value.

**Results:**

Plasma GSTα protein levels were significantly lower in the AD group than in the CN group (0.94 vs1.05, *p* = 0.04) and the MCI group (0.94 vs1.09, *p* < 0.001). Plasma GSTα protein level changes were positively correlated with altered MMSE levels in MCI and AD patients (*r* = 0.09, *p* = 0.04). The AUC (95% CI) of the area under the prediction curve of plasma GSTα protein levels for MCI was 0.63 (0.54–0.71), *p* = 0.02, and the AUC (95% CI) of the area under the prediction curve of plasma GSTα protein levels for AD was 0.74 (0.69–0.80), *p* < 0.001. At the same time, we plotted ROC curves for the difference in the change of plasma GSTα protein levels after 1 year of follow-up. The results showed that the AUC (95% CI) of the area under the prediction curve of plasma GSTα protein levels change for MCI was 0.76 (0.696–0.83), *p* < 0.001, and the AUC (95% CI) of the area under the prediction curve of plasma GSTα protein levels change for AD was 0.75 (0.69–0.80), *p* < 0.001.

**Conclusion:**

The findings of the study indicated notable differences in GSTα protein levels among patients with MCI and those with AD after a one-year follow-up period. Furthermore, a positive correlation was observed between changes in GST αprotein levels and the decline in both baseline and cognitive function. This suggests that GSTα protein could potentially act as a biomarker for both MCI and AD, offering fresh insights for early detection and intervention strategies.

## 1 Introduction

With the aging population, the prevention and treatment of Alzheimer’s disease (AD) are increasingly becoming a global concern (Sexton et al., 2022; GBD 2016 Dementia Collaborators, 2019). As a common neurodegenerative disease, AD places a heavy burden on patients and their families (Livingston et al., 2017; Livingston et al., 2020). Mild cognitive impairment(MCI), on the other hand, is considered a transitional stage between normal aging and AD, and early recognition and intervention of mild cognitive impairment is essential to slow down the development of AD (Wolfova et al., 2021; Malik et al., 2024).

Glutathione S-transferase (GST) is an important detoxifying enzyme that protects cellular health by counteracting aging-related oxidative and chemical stress (Tchaikovskaya et al., 2005). Oxidative stress is widely recognized as a critical pathogenic factor in the pathogenesis of MCI and AD (Ansari et al., 2023; Ibrahim, 2020; Alamro et al., 2020; Zheng et al., 2022). Oxidative stress not only leads to neuronal cell damage and apoptosis but also has the potential to have profound effects on changes in cognitive function (Ibrahim, 2020; Choi et al., 2023). Glutathione S-transferase omega-1 (GSTO1) is closely associated with the pathogenesis of Alzheimer’s disease (AD) (Capurso et al., 2010; Li et al., 2003; Jia et al., 2022). Furthermore, recent studies have indicated that the glutathione S-transferase omega-1 and omega-2 genes (GSTO1, GSTO2) may elevate the risk of AD in older adults by reducing the impact of oxidative stress (Allen et al., 2012).

Glutathione S-transferase alpha (GSTα) plays an essential physiological role *in vivo*. Conversely, variations in GSTα activity could indicate the level of oxidative stress present in the body, which is crucial in the progression of neurodegenerative disorders. Additionally, GSTα might influence the functionality and longevity of neuronal cells by engaging in particular metabolic pathways. Therefore, an in-depth exploration of the dynamic changes of GSTα in MCI and AD will not only enhance our understanding of the pathomechanisms of these diseases but also provide new biomarkers for early intervention, which will improve the quality of life of elderly patients. By studying the dynamic changes of GSTα in MCI and AD, we may be able to open up new directions for the treatment and prevention of AD.

## 2 Methods

### 2.1 Study population

The data used in the present study were from the online database of the Alzheimer’s Disease Neuroimaging Initiative (ADNI).^1^ Healthy individuals (as control 54), patients with mild cognitive impairment (345), and patients with AD (96) were followed up for 1 year, and data on dynamic changes in GSTα protein levels in plasma and primary information data (including clinical assessment, neuropsychological tests) were collected.

#### 2.1.1 Inclusion criteria

Cognitive status: subjects should be classified as normal cognition (CN), mild cognitive impairment (MCI), and Alzheimer’s disease (AD) according to the ADNI protocol.

Physical health: participants should have no major health problems such as serious heart disease, liver disease, kidney disease, or malignancy that could affect the study results.

Consent to participate in the study: subjects or their legal representatives must sign an informed consent form.

#### 2.1.2 Exclusion criteria

Non-compliance with cognitive status criteria: subjects should be excluded if their cognitive status does not meet the prescribed CN, EMCI, LMCI, or AD criteria.

Inability to provide consent: subjects who fail to provide informed consent or are legally restricted from participating in the study.

No GSTα data available: individuals whose plasma GSTα protein levels are not available through laboratory testing should be excluded from consideration.

### 2.2 Diagnosis of MCI and AD

Recruitment methods and diagnostic approaches for ADNI have been reported previously (Allen et al., 2012). Individuals diagnosed with mild AD were required to fulfill the criteria established by the National Institute of Neurological and Communicative Disorders and Stroke–Alzheimer’s Disease and Related Disorders Association for probable Alzheimer’s Disease (Kiely et al., 2018).

### 2.3 Standard protocol approvals, registrations, and patient consents

Written consent from participants was acquired in every source study by the Declaration of Helsinki and with the approval of local Institutional Review Boards. Comprehensive information regarding ethics approval, study design, participant recruitment, and clinical assessments can be found at adni-info.org.

### 2.4 Statistical methods

The Kolmogorov–Smirnov test was employed to assess the normality of the data. Data that adhered to a normal distribution were reported as the mean ± standard deviation. For comparisons between two groups, the two-sample t-test was applied, while a one-way ANOVA was utilized for comparisons involving three groups. In cases where the data did not meet the assumptions of normality, the median was reported alongside the first and third quartiles; in these instances, the Mann–Whitney U test was used for two-group comparisons, and the Kruskal-Wallis test was applied for three groups. Categorical data were represented as percentages, with the χ^2^ test implemented for analysis. Pearson’s correlation coefficient was used for normally distributed data, whereas Spearman’s correlation analysis was applied to examine relationships among non-normally distributed parameters. Receiver operating characteristic (ROC) curves were generated to evaluate GSTα protein levels in the context of mild cognitive impairment (MCI) and Alzheimer’s disease (AD), and the area under the curve (AUC) was computed. All hypothesis tests were conducted as two-tailed, with a significance threshold set at *p* < 0.05. Data processing and graphical representation were performed using SPSS version 26.0 and Origin 2021 software.

## 3 Results

### 3.1 Essential characteristics of the study population

A total of 495 subjects were included in this study, and the essential information is shown in Table 1. There were significant differences in MMSE and CDR scores and the type of APOE genes carried by CN, MCI, and AD before follow-up (*p* < 0.05), and there were no significant differences in gender, age, years of education, and GSTα, (*p* > 0.05).

**Table 1:** Characteristics of participants.

Characteristics	CN (*N* = 54)	MCI (*N* = 345)	AD (*N* = 96)	*X*^2^, H	*p*-value
Age, median (IQR), years	73.20 (71.4, 79.2)	75.30 (70.5, 80.4)	76.60 (70.9, 80.6)	0.251	0.882
Female, *n* (%)	26 (48.1%)	121 (35.1%)	43 (44.8%)	5.443	0.0658
Education, median (IQR), years	16.00 (13.0, 18.0)	16.00 (14.0, 18.0)	16.00 (13.0, 18.0)	2.926	0.232
APOE4 0	49 (90.7%)	160 (46.4%)	32 (33.3%)	50.425	<0.001
1	5 (9.3%)	142 (41.2%)	44 (45.8%)		
2	0 (0%)	43 (12.5%)	20 (20.8%)		
MMSE Scores, median (IQR)	29.00 (29.0, 30.0)	27.00 (26.0, 29.0)	24.00 (22.0, 25.0)	204.318	<0.001
CDR, median (IQR)	0.00 (0.0, 0.0)	1.50 (1.0, 2.0)	4.00 (3.5, 5.0)	283.954	<0.001
GST-α, median (IQR), ng/ml	1.20 (1.0, 1.4)	1.11 (0.9, 1.3)	1.15 (0.9, 1.3)	4.281	0.118

Measurement data are represented by *n* (%); numeration data are represented by median (IQR).

### 3.2 Dynamics of plasma GSTα at 1 year of follow-up

**Figure 1:**
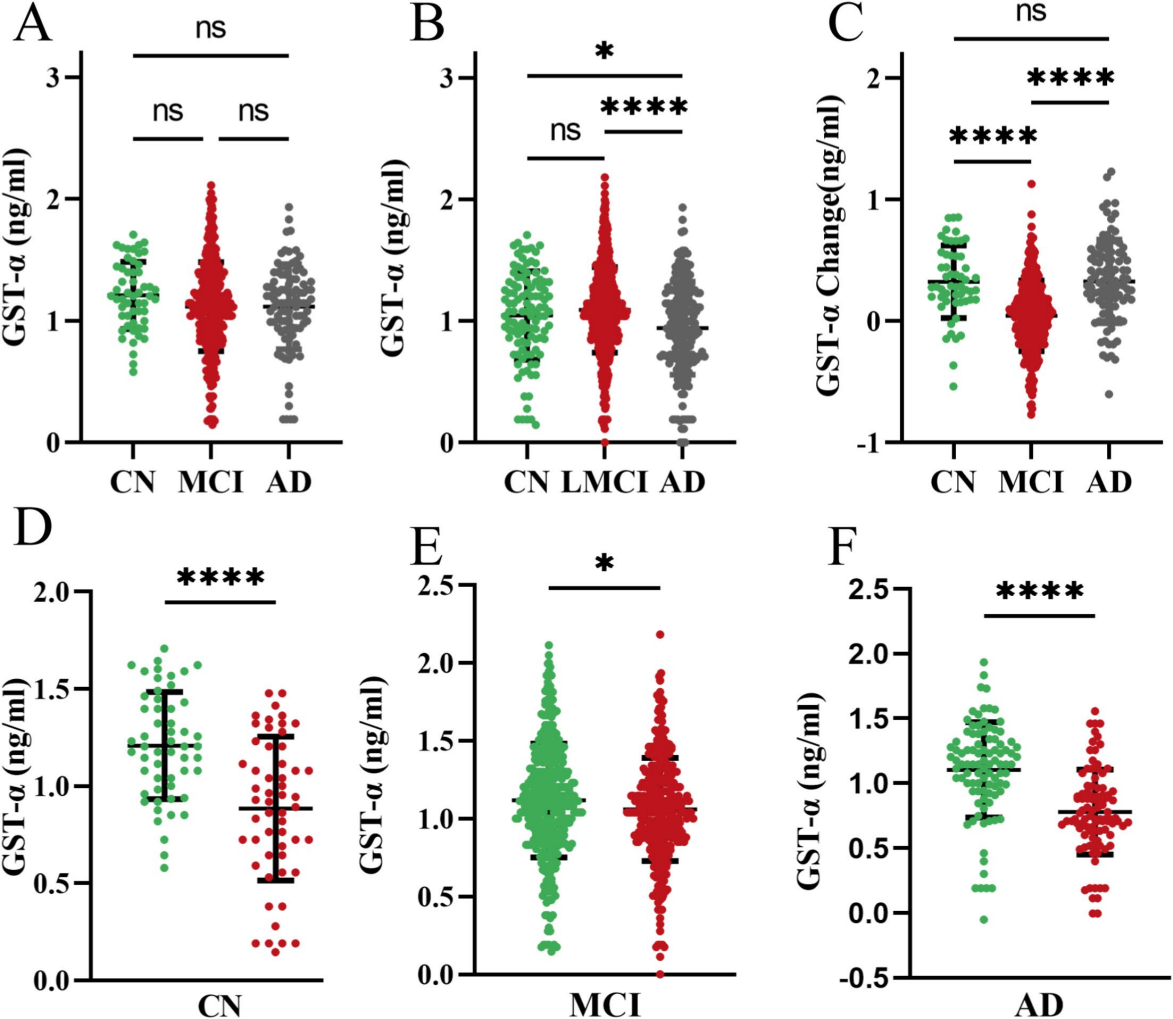
Dynamics of plasma GSTα at 1 year of follow-up. **(A)** Levels of GSTα at baseline at follow-up in the three cohorts. **(B)** Total GSTα levels after 1 year of follow-up in the three cohorts. **(C)** Changes in GSTα from baseline after 1 year of follow-up in the three cohorts. **(D)** Difference between GSTα at 1 year of follow-up and baseline in the control group. **(E)** Difference between GSTα at 1 year of follow-up and baseline in the MCI group. **(F)** Difference between GSTα at 1 year of follow-up and baseline in the AD group. The error bars are the SEM, *p < 0.05, **p < 0.01, ***p < 0.001, ****p < 0.0001. CN, control group, AD, Alzheimer’s disease patient group; MCI, mild cognitive impairment patient group.

The first measurement plasma GSTα protein levels at follow-up were not statistically different among the three groups (*p* > 0.05, Figure 1A). A year later, plasma protein levels of GSTα in the Alzheimer’s Disease (AD) group were significantly lower than those in the Cognitively Normal (CN) group (0.94 vs. 1.05, *p* = 0.04) and the Mild Cognitive Impairment (MCI) group (0.94 vs. 1.09, *p* < 0.001) (Figure 1B). The difference in GSTα changes in plasma protein levels after 1 year of follow-up was minimal when comparing the MCI group to the AD group (0.04 vs. 0.33, *p* < 0.001) (Figure 1C). Additionally, plasma GSTα protein levels were lower than baseline after 1 year of follow-up in the CN group (1.21 vs. 0.88, *p* = 0.04) (Figure 1D), in the MCI group (1.12 vs. 1.06, *p* = 0.03) (Figure 1E), and in the AD group (1.11 vs. 0.78, *p* < 0.001) (Figure 1F).

### 3.3 Correlation of plasma GSTα protein levels dynamics with baseline GSTα and cognition

**Figure 2:**
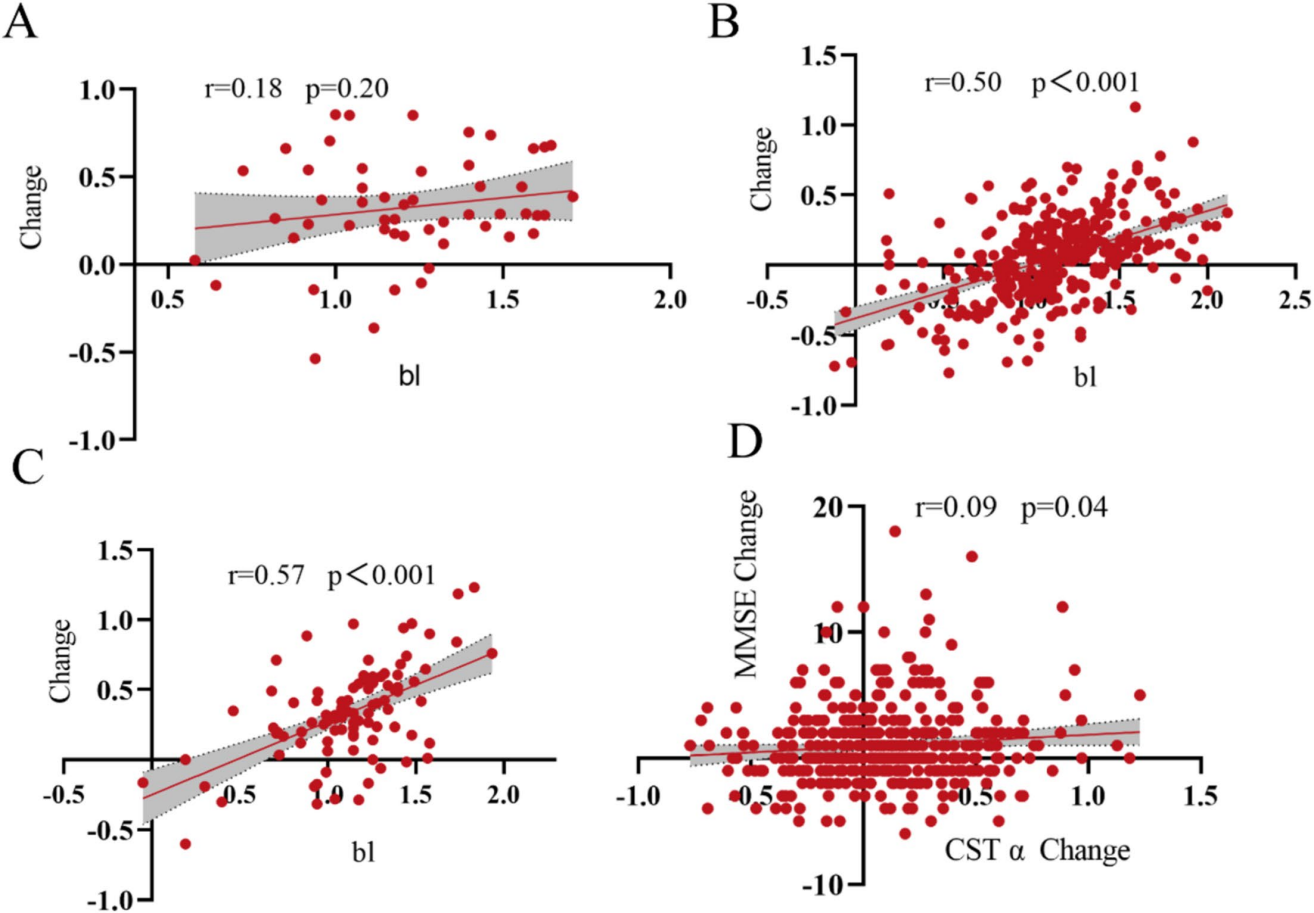
Correlation of GSTα changes with baseline. **(A)** Correlation of GSTα changes from baseline in the control group. **(B)** Correlation of GSTα changes with baseline in the MCI group. **(C)** Correlation of GSTα changes with baseline in the AD group. **(D)** Correlation of GSTα changes with MMSE change in the MCI and AD group.

We analyzed the relationship between GSTα protein levels changes and baseline in the plasma three populations, with a non-significant correlation between plasma GSTα protein levels changes and baseline levels in the CN group (*r* = 0.18, *p* = 0.20) (Figure 2A) and the MCI group plasma GSTα changes were positively correlated with baseline levels (*r* = 0.50, *p* < 0.001) (Figure 2B), and in the AD group (*r* = 0.57, *p* < 0.001) (Figure 2C). At the same time, Plasma GSTα protein level changes were positively correlated with altered MMSE levels in MCI and AD patients (*r* = 0.09, *p* = 0.04) (Figure 2D).

### 3.4 Plasma GSTα protein levels predict AD and MCI values

**Figure 3:**
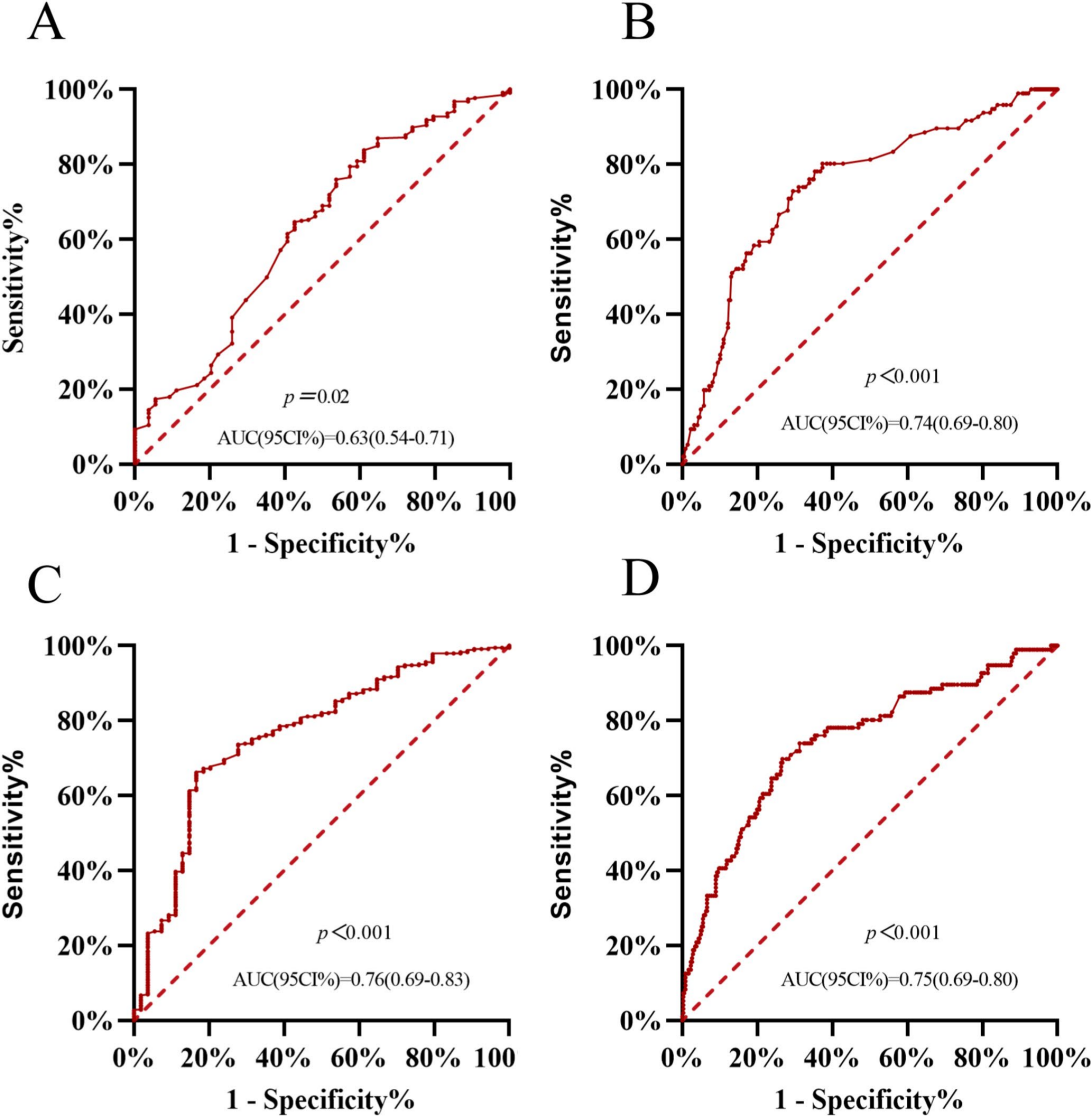
Plasma GSTα predicts AD and MCI values. **(A)** Predictive ability of ROC curve analysis of GSTα to differentiate between CN and MCI patients. **(B)** Predictive ability of ROC curve analysis of GSTα to distinguish between AD and MCI patients. **(C)** Predictive Power of ROC Curve Analysis of GSTα Alterations to Distinguish between CN and MCI Patients at 1-Year Follow-Up. **(D)** Predictive Power of ROC Curve Analysis of GSTα Alterations to Distinguish Between AD and MCI Patients at 1-Year Follow-Up. CN, control group; AD, Alzheimer’s disease patient group; MCI, mild cognitive impairment patient group.

To evaluate the ability of plasma GSTα to predict MCI and AD, we plotted ROC curves, and after 1 year of follow-up, the AUC of the area under the curve of plasma GSTα for MCI prediction (95% CI) was 0.63 (0.54–0.71), *p* = 0.02, (Figure 3A), and that of the area under the curve of plasma GSTα for AD prediction (95% CI) was 0.74 (0.69–0.80), *p* < 0.001 (Figure 3B), while we plotted ROC curves for the difference in plasma GSTα changes after 1 year of follow-up, and the results showed that plasma GSTα alteration on MCI predicted area under the curve AUC (95% CI) 0.76 (0.696–0.83), *p* < 0.001, (Figure 3C), plasma GSTα alteration on AD predicted area under the curve AUC (95% CI) 0.75 (0.69–0.80), *p* < 0.001 (Figure 3D), and plasma GSTα alteration had diagnostic value and accurate risk measurement ability for MCI and AD.

### 3.5 Subgroup analysis of plasma GSTα protein levels alterations to predict AD and MCI value

**Figure 4:**
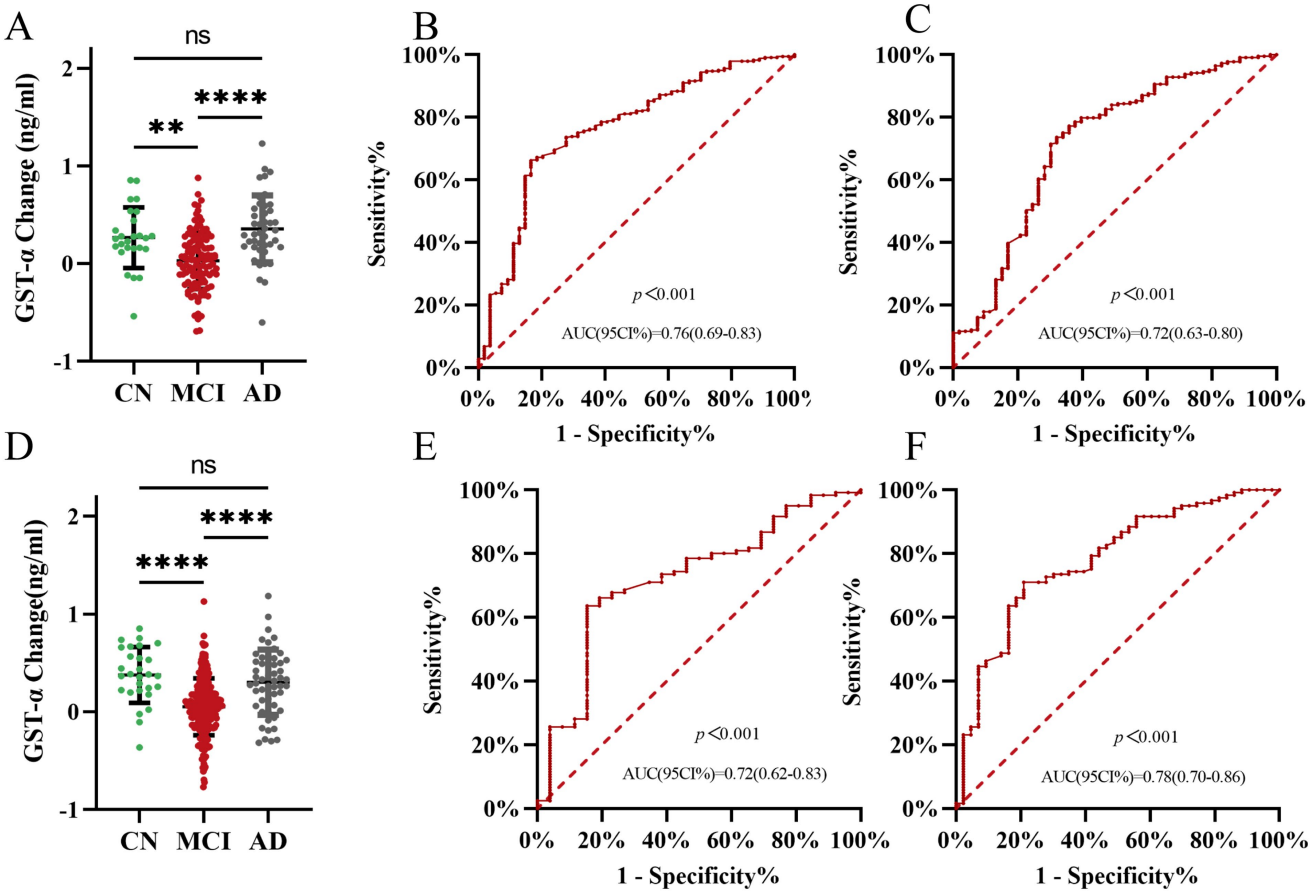
Subgroup analysis of the ability of plasma GSTα to predict AD and MCI. **(A)** Changes in GSTα after 1 year of follow-up in the man cohorts. **(B)** Predictive ability of ROC curve analysis of GSTα Alterations to Distinguish between CN and MCI patients in the male cohorts at 1-year Follow-Up. **(C)** Predictive ability of ROC curve analysis of GSTα Alterations to Distinguish between AD and MCI patients in the male cohorts at 1-year Follow-Up. **(D)** Changes in GSTα after 1 year of follow-up in the female cohorts. **(E)** Predictive ability of ROC curve analysis of GSTα alterations to distinguish between CN and MCI patients in the female cohorts at 1-year Follow-Up. **(F)** Predictive ability of ROC curve analysis of GSTα Alterations to Distinguish between AD and MCI patients in the female cohorts at 1-year Follow-Up. The error bars are the SEM, *p < 0.05, **p < 0.01, ***p < 0.001, ****p < 0.0001.CN, control group; AD, Alzheimer’s disease patient group; MCI, mild cognitive impairment patient group.

We statistically analyzed the difference in plasma GSTα protein level changes by gender at 1-year follow-up. The results showed that plasma GSTα changes were less than MCI changes in men than in the AD group (0.03 vs. 0.36, *p* < 0.001) (Figure 4A), and plasma GSTα changes were less than MCI changes in women than in the AD group (0.05 vs. 0.30, *p* < 0.001) (Figure 4D). The ROC curves were plotted, and the results showed that plasma GSTα alteration in males had an AUC (95% CI) of 0.76 (0.69–0.83), *p* < 0.001, for the area under the prediction curve for MCI (Figure 4B), and plasma GSTα alteration had an AUC (95% CI) of 0.72 (0.63–0.80), *p* < 0.001, for the area under the prediction curve for AD (Figure 4C). Plasma GSTα alterations in women had an AUC (95% CI) of 0.72 (0.62–0.83), *p* < 0.001, for the area under the prediction curve for MCI (Figure 4E), and plasma GSTα alterations had an AUC (95% CI) of 0.78 (0.70–0.86), *p* < 0.001, for the area under the prediction curve for AD (Figure 4F), and plasma GSTα alterations in different sexes had the same diagnostic value and accurate risk-measurement ability for MCI and AD.

## 4 Discussion

In this research, we investigated the fluctuating levels of glutathione S-transferase alpha (GSTα) in individuals with mild cognitive impairment (MCI) and Alzheimer’s disease (AD) and explored its connection to cognitive abilities through data analysis from the Alzheimer’s Disease Neuroimaging Initiative (ADNI) database. The findings indicated that there was a significant difference in GSTα protein levels among MCI and AD patients after a year of observation, with alterations in GSTα showing a negative correlation with cognitive function decline. This evidence implies that GSTα protein levels could act as a potential biomarker for MCI and AD, offering new perspectives for early detection and intervention.

To begin with, the findings of this current research align with earlier studies and reinforce the significance of GST in conditions related to neurodegeneration (Dasari et al., 2018; Kumar et al., 2017). Three previous innovative brain network analysis models (DGCL, PALH, and BSFL) have improved the efficiency of brain network construction, the accuracy of disease prediction, and the fusion of structural and functional features, providing strong support for neuroscience research and early treatment of cognitive disorders (Zong et al., 2024; Zuo et al., 2024; Zuo et al., 2023). GSTα, a vital antioxidant enzyme, plays an important role in protecting against oxidative stress (Marugame et al., 2022). In the pathogenesis of MCI and AD, oxidative stress is considered an important causative factor. Studies have shown that changes in the expression and activity of GSTα are closely associated with the development of various neurodegenerative diseases (Grabska-Kobyłecka et al., 2023; Wang, 2015). In the present study, we found significant differences in GSTα protein levels between MCI and AD patients after 1 year of follow-up, suggesting that GSTα protein levels may change more rapidly in the later stages of the disease.

This study also found that changes in GSTα protein levels were positively correlated with the deterioration of cognitive function. However, the correlation coefficient was small, possibly due to the short follow-up time and mental decline, which is a slow and complex process. Still, the ROC curve analysis, the area under the curve line of the GSTα level, and its changes suggest that the GSTα can be used as a protein to assess the cognitive function of patients. Decreased levels of GSTα are associated with a gradual decline in cognitive function. While this study primarily examined the fluctuations of GSTα in plasma, upcoming research could investigate its expression and activity in both the brain and neuronal cells to attain a deeper insight into the true function of GSTα within the nervous system.

This study did not specifically look at patients who converted from MCI to AD. Future studies should expand the sample size and design more detailed follow-up protocols to analyze changes in GSTα expression levels in these converted patients, thereby validating the potential of GSTα protein levels as a prognostic marker. Although this study found changes in the expression level of GSTα in the MCI group, it did not make a clear distinction between patients who progressed to AD and those who did not. Future studies should use more refined analytical methods, such as multifactor regression analysis, in conjunction with other biomarkers to assess the prognostic value of GSTα protein levels in the progression of MCI to AD.

In conclusion, by analyzing data from the ADNI database, this study initially revealed the dynamic changes of GSTα protein levels in MCI and AD and its correlation with cognitive function. Thereby providing new ideas for early detection and progression monitoring of MCI and AD.

## Data Availability

Publicly available datasets were analyzed in this study. This data can be found at: ADNI; www.loni.ucla.edu/ADNI.

## Footnotes

^1^ www.loni.ucla.edu/ADNI
